# Association of cigarette smoking, smoking cessation with the risk of cardiometabolic multimorbidity in the UK Biobank

**DOI:** 10.1186/s12889-024-19457-y

**Published:** 2024-07-16

**Authors:** Shuo Zhang, Zhou Jiang, Hao Zhang, Yuxin Liu, Jike Qi, Yu Yan, Ting Wang, Ping Zeng

**Affiliations:** 1 Department of Biostatistics, School of Public Health, Xuzhou Medical University, Xuzhou, 221004 Jiangsu China; 2 Jiangsu Engineering Research Center of Biological Data Mining and Healthcare Transformation, Xuzhou Medical University, Xuzhou, 221004 Jiangsu China

**Keywords:** Cigarette smoking, Smoking cessation, UK Biobank, Cardiometabolic multimorbidity, Multi-state model

## Abstract

**Background:**

To investigate the association between cigarette smoking, smoking cessation and the trajectory of cardiometabolic multimorbidity (CMM), and further to examine the association of age at smoking initiation and smoking cessation with CMM.

**Methods:**

This study included 298,984 UK Biobank participants without cardiometabolic diseases (CMDs) (including type 2 diabetes, coronary heart diseases, stroke, and hypertension) at baseline. Smoking status was categorized into former, current, and never smokers, with age at smoking initiation and smoking cessation as a proxy for current and former smokers. The multi-state model was performed to evaluate the association between cigarette smoking, smoking cessation and CMM.

**Results:**

During a median follow-up of 13.2 years, 59,193 participants developed first cardiometabolic disease (FCMD), 14,090 further developed CMM, and 16,487 died. Compared to former smokers, current smokers had higher risk at all transitions, with hazard ratio (95% confidence interval) = 1.59 (1.55 ∼ 1.63) vs. 1.18 (1.16 ∼ 1.21) (*P* = 1.48 × 10^− 118^) from health to FCMD, 1.40 (1.33 ∼ 1.47) vs. 1.09 (1.05 ∼ 1.14) (*P* = 1.50 × 10^− 18^) from FCMD to CMM, and 2.87 (2.72 ∼ 3.03) vs. 1.38 (1.32 ∼ 1.45) (*P* < 0.001) from health, 2.16 (1.98 ∼ 2.35) vs. 1.25 (1.16 ∼ 1.34) (*P* = 1.18 × 10^− 46^) from FCMD, 2.02 (1.79 ∼ 2.28) vs. 1.22 (1.09 ∼ 1.35) (*P* = 3.93 × 10^− 17^) from CMM to death; whereas quitting smoking reduced the risk attributed to cigarette smoking by approximately 76.5% across all transitions. Reduced risks of smoking cessation were also identified when age at quitting smoking was used as a proxy for former smokers.

**Conclusions:**

Cigarette smoking was associated with a higher risk of CMM across all transitions; however, smoking cessation, especially before the age of 35, was associated with a significant decrease in CMM risk attributed to cigarette smoking.

**Supplementary Information:**

The online version contains supplementary material available at 10.1186/s12889-024-19457-y.

## Background

Cigarette smoking is a major behavioral risk factor with a leading attributable health burden worldwide, and is a long-standing challenge for public health [1, 2]. Existing studies have well-documented the strong association between cigarette smoking and the occurrence of many diseases, including lung cancer, cardiovascular disease, and chronic respiratory disease [2–4]. Cigarette smoking is also the major cause of premature death around the world, accounting for more than 5 million or 12% of all-cause deaths annually [5, 6].

Fortunately, epidemiological studies have demonstrated that smoking cessation is significantly associated with a reduced risk of disease incidence and death [7–10]. For instance, cigarette smoking can directly damage heart, with the more people smoking the worse their heart function; however, the function of heart can be restored or even reversed to some extent after quitting smoking [4]. Cigarette smoking is significantly related to an increased risk of type II diabetes (T2D), but the risk may decrease to the level of never smokers after 10 years of smoking cessation [11]. Cigarette smoking also substantially increases the risk of coronary heart diseases (CHD) [7], but the risk of CHD would be halved after one year of quitting smoking [3], and the risk would even fall significantly to the level of never smokers after 15 years of smoking cessation [12]. Similar risk reductions are also observed for patients of stroke and hypertension when stopping smoking [13, 14].

Further, previous studies have discovered that, compared to never smokers, both former and current smokers had a significantly higher risk of multimorbidity, all-cause mortality, and multimorbidity-related mortality [15, 16]. Studies have shown that approximately half of regular smokers who start smoking before age 18 will die of tobacco-related diseases, losing an average of 10 years of life unless they quit smoking permanently [17–19]. In contrast, quitting smoking before the age of 30 was an important health behavior, helping increase life expectancy by 8–9 years [20].

With the rising proportion of aging people globally, the prevalence of cardiometabolic multimorbidity (CMM), defined as the co-occurrence of at least two cardiometabolic diseases (CMDs) (including T2D, CHD, stroke, and hypertension in our study) [21, 22] has been increasing rapidly [21, 23]. CMM is becoming a global health burden due to its association with reduced quality of life, greater use of healthcare resources, and higher risk of disability and death [24, 25]. It has been shown that, compared to individual CMDs, the accumulation of CMM multiply elevates the risk of death and significantly reduces life expectancy [21, 26], the aging population makes this issue much worse [21, 22].

Although there is already compelling evidence that cigarette smoking is closely associated with the development of individual CMDs, to our knowledge, few existing studies to date have independently investigated the association of cigarette smoking and smoking cessation with CMM. Investigations of the association between age at smoking initiation and smoking cessation and CMM are even more lacking, and disease trajectories have not been examined. To fill in these knowledge gaps, we here analyzed the large-scale prospective UK Biobank cohort data (*N* = 298,984) [27] to evaluate the effects of cigarette smoking and smoking cessation on the trajectory of CMM, including the transition from no CMD to first cardiometabolic disease (FCMD) and then to CMM and further to death.

## Methods

### Study participants

We conducted our analyses in the UK Biobank, a large-scale population-based prospective study with approximately half a million participants aged 40–69 years, which collected an unprecedented amount of biological and medical data resource since 2006 [27]. In this study, we filtered out participants who met any of the below criteria at recruitment: (i) withdrew from the survey; (ii) missed data on smoking status; (iii) had type I diabetes or gestational diabetes; (iv) suffered from any CMDs before recruitment. Afterwards, 298,984 participants without any CMDs at the time of recruitment were initially included.

### Outcome identification

Following previous studies [21, 28, 29], CMM was defined as co-occurrence of at least two of the four focused CMDs (i.e., T2D, CHD, stroke, and hypertension). In the UK Biobank, participants′ data were collected from self-reported diseases diagnosed by doctors and hospital inpatient records. The diagnoses were coded according to the International Classification of Diseases, 9th Revision (ICD-9) and 10th Revision (ICD-10), and the Office of Population Censuses and Surveys Classification of Interventions and Procedures, version 4 (OPCS-4) (Table S1). The date of occurring CMM was the earliest date of occurrence for any of the four CMDs. The incident cases of all-cause death were identified through linking to national death registries.

### Assessment of cigarette smoking and smoking cessation

Detailed information regarding cigarette smoking and smoking cessation was obtained from touch screen questionnaires [27]. Participants were categorized according to smoking status and amount smoked (Table S2). Specifically, in terms of smoking status defined in prior studies [30, 31], participants were considered as never smokers if they had not smoked at least 100 cigarettes in their lifetime. Participants who had smoked at least 100 cigarettes in their lifetime were treated as ever smokers, with those who had quit smoking before recruitment considered as former smokers, and those who had not quit smoking deemed as current smokers.

Furthermore, the baseline pack-years of smoking were calculated as the average number of cigarettes smoked per day, which can be treated as continuous and categorical variables (all participants were categorized as “None”, “Up to 10”, “Between 11 and 20”, “Between 21 and 30”, and“More than 30”) [32, 33]. Then, former smokers were classified into various groups regarding age at quitting smoking [30] and current smokers were classified into various groups regarding age at starting smoking (see below) [18], and both the two types of smoking behaviors were analyzed to evaluate the association between smoking cessation and CMM.

### Measurement of potential covariates

Several sociodemographic characteristics and lifestyle factors were included as covariates to adjust for their potential influences, including age, sex, race, education levels, household income [34], body mass index (BMI), alcohol consumption, physical activity levels [35], systolic blood pressure (SBP), and healthy diet score [36]. Treatment details of some covariates were further described in the Supplementary File. Missing values of covariates were imputed by the method of multivariate imputation by chained equations via the R MICE package [37].

### Statistical analysis

#### Association of cigarette smoking, smoking cessation with CMM

**Fig. 1:**
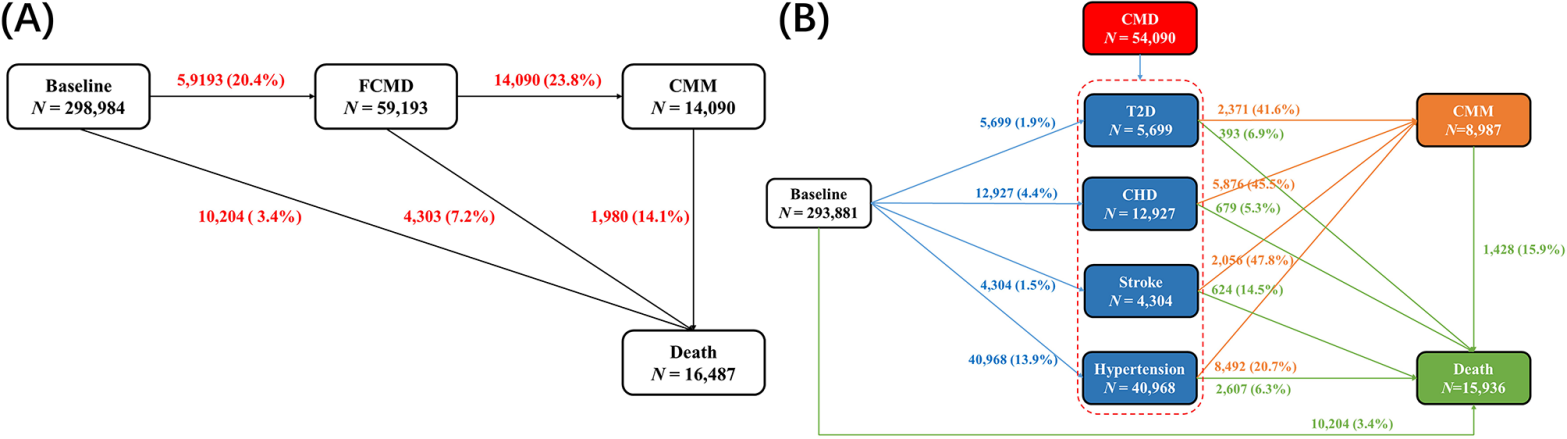
(**A**) Numbers (percentages) of participants in transition pattern A from baseline to FCMD, CMM, and death. (**B**) Numbers (percentages) of participants in transition pattern B from baseline to one of T2D, CHD, stroke, and hypertension, then to CMM, and subsequently to death. CMDs include T2D, CHD, stroke, and hypertension. CMM is defined as the co-occurrence of at least two of the above-mentioned diseases. T2D: type II diabetes, CHD: coronary heart diseases, FCMD: first cardiometabolic disease, CMM: cardiometabolic multimorbidity

Cox proportional hazards regression was applied to investigate the associations of cigarette smoking and smoking cessation with CMM. Schoenfeld′s residuals were applied to test the proportional hazards assumption of Cox model [38], but no violations were observed. We employed the multi-state model to assess the effects of cigarette smoking and smoking cessation on the temporal disease progression from baseline to FCMD, CMM, or death [39]. The multi-state model was a powerful extension of the competing risk model for exploring the influence of certain factors on different stages of the process. We constructed five transitions and referred them to as pattern A (Fig. 1A): (i) from baseline to FCMD; (ii) from FCMD to CMM; (iii) from baseline to death; (iv) from FCMD to death from any cause; (v) from CMM to death from any cause.

At each transition stage, the covariates mentioned above were incorporated into the model, with hazard ratio (HR) and its 95% confidence interval (CI) reported. When modeling the transition to FCMD, the follow-up time was calculated from the date of recruitment to the date of diagnosis of FCMD, the date of death, or the end of the follow-up (31 Dec, 2021), whichever occurred first. When modeling the transition to CMM, the follow-up time was calculated from the date of the subsequent second event, the date of death, or the end of the follow-up (31 Dec, 2021), whichever came first.

To further evaluate the association between cigarette smoking, smoking cessation and CMM, we initially utilized age at starting smoking (< 18 years or ≥ 18 years) as a proxy for current smoker, and conducted the above analyses. Subsequently, we employed age at quitting smoking (< 35 years, 35–44 years, 45–54 years, or ≥ 55 years) as a proxy for former smoker [30] and replicated the analyses. Additionally, we divided former and current smokers into low pack-years (< 20) and high pack-years (≥ 20) and extended the above analyses [40].

#### Association of cigarette smoking, smoking cessation with four FCMDs

We also employed the multi-state model with the same setting to estimate the effects of cigarette smoking and smoking cessation on different pathways from baseline health to CMM or to death. We here divided CMM into four individual CMDs (i.e., T2D, CHD, stroke, and hypertension), resulting in a total of fourteen transition stages which were referred to as pattern B (Fig. 1B). In this pattern, to obtain more affluent information about the association of smoking cessation with individual CMDs, and to produce statistically reliable estimates with a sufficient number of cases during each transition, former smokers were only categorized into two groups in terms of quitting smoking before and after the age of 45 years [30]. Furthermore, if a participant was diagnosed with at least two of CMDs on the same date, we could not ascertain the temporal sequence of disease occurrences and thus excluded them here, leaving 293,881 participants.

#### Subgroup analyses stratified by covariates

To evaluate the robustness and potential variation of our findings, we performed several subgroup analyses. Briefly, we repeated the analyses in sub-studies stratified by sex (male or female), income (<£31,000 or ≥£31,000) [34], TDI (less or larger than the median), alcohol consumption (drinker or non-drinker), physical activity level (low, moderate, or high), healthy diet score (0–2 or 3–5), and BMI (< 25, 25–30, or ≥ 30 kg/m^2^).

Further, we carried out sensitivity analyses only in white participants, and excluded cases/deaths that occurred within two years prior to follow-up and participants who quit smoking within two years before and after recruitment to reduce possible reverse causality [41, 42]. We also excluded smokers who stopped smoking due to illness/disease and those who quit smoking due to physician recommendation (filed 6157) to avoid reverse causality [30].

### Estimation of attributable fractions and population-attributable fractions

To further quantify the associations between cigarette smoking and smoking cessation with CMM, we estimated the attributable fraction percent (AF%) and population-attributable fraction percent (PAF%).

$${\text{AF\% }}\;=\;\frac{{{\text{HR}}\; - \;1}}{{{\text{HR}}}} \times {\text{100\% , }}\;{\text{PAF\% }}\;=\;\frac{{{P_e}({\text{HR}}\; - \;1)}}{{{P_e}({\text{HR}}\; - \;1)\;+\;1}} \times 100\%$$

where *P*_*e*_ indicates the proportion of ever smokers (both former and current smokers) obtained from the exposure-specific UK Biobank dataset, and the HR is the risk estimate of ever smokers vs. never smokers.

The percentage of increased risk of CMM incidence and death avoided by quitting smoking (compared with continuing to smoke) was estimated by (HR^a^-HR^b^)/(HR^a^-1), where HR^a^ is the risk of current vs. never smokers and HR^b^ is the risk of former vs. never smokers [30].

All statistical analyses were performed under the R software computing environment (version 4.3.1), and a two-sided *P* < 0.05 was considered statistically significant.

## Results

### Participant characteristics

There were a total of 298,984 participants without any CMD at recruitment. The summary information of all included participants under different conditions was described in Table 1. Particularly, among current smokers, the average age was 53.6 ± 8.1 years; among them, 50.4% were males and the average age was 53.6 ± 8.1 years, and 49.6% were females and the average age was 53.6 ± 7.9 years. Among former smokers, the average age of quitting smoking was 38.5 ± 11.4 years; among them, 47.4% males quit smoking, with an average age of quitting smoking was 38.5 ± 11.4 years, and females accounted for 52.6% of former smokers, with an average age of 38.5 ± 11.3 years.

**Table 1:** Summary information of all included participants in the UK Biobank cohort under different statuses

covariates	without FCMD (*N* = 239,791)	T2D (*N* = 8,040)	CHD (*N* = 16,185)	stroke (*N* = 4,965)	hypertension (*N* = 45,883)	CMM (*N* = 14,090)
Smoking status (%)						
Never	162,898 (67.9)	4,305 (53.5)	8,770 (54.2)	2,759 (55.6)	26,964 (58.8)	7,407 (52.6)
Former	53,988 (22.5)	2,365 (29.4)	4,821 (29.8)	1,395 (28.1)	13,260 (28.9)	4,357 (30.9)
Current	22,638 (9.4)	1,355 (16.9)	2,576 (15.9)	803 (16.2)	5,605 (12.2)	2,306 (16.4)
Age at starting smoking, years (SD)	17.4 ± 4.4	17.0 ± 4.9	17.1 ± 4.6	17.4 ± 4.6	17.2 ± 4.6	17.1 ± 4.7
Age at starting smoking (%)						
< 18	9,633 (4.0)	479 (6.0)	964 (6.0)	341 (6.9)	2,252 (4.9)	876 (6.2)
≥ 18	13,050 (5.4)	876 (10.9)	1,612 (10.0)	462 (9.3)	3,353 (7.3)	1,430 (10.1)
Age at quitting smoking, years (SD)	38.3 ± 11.7	42.7 ± 12.6	41.8 ± 13.0	42.5 ± 4.6	41.5 ± 12.7	42.6 ± 12.9
Age at quitting smoking (%)						
< 35	22,921 (9.6)	682 (8.5)	1,594 (9.8)	433 (8.7)	4,401 (9.6)	1,302 (9.2)
35–44	16,112 (6.7)	626 (7.8)	1,268 (7.8)	387 (7.8)	3,698 (8.1)	1,211 (8.6)
45–54	10,017 (4.2)	609 (7.6)	1,124 (6.9)	308 (6.2)	2,995 (6.5)	1,044 (7.4)
≥ 55	4,938 (2.1)	448 (5.6)	817 (5.0)	267 (5.4)	2,166 (4.7)	800 (5.7)
Pack-years (SD)	19.2 ± 16.1	27.3 ± 21.4	25.6 ± 20.3	26.4 ± 21.2	24.5 ± 19.7	27.0 ± 21.1
Age, years (SD)	54.3 ± 8.0	57.5 ± 7.9	59.1 ± 7.3	60.2 ± 7.2	58.8 ± 7.5	59.6 ± 7.2
BMI, kg/m^2^ (SD)	26.3 ± 4.2	30.7 ± 5.5	27.6 ± 4.4	27.0 ± 4.5	28.2 ± 4.9	28.8 ± 5.1
TDI (SD)	-1.5 ± 3.0	-0.4 ± 3.4	-1.2 ± 3.2	-1.2 ± 3.2	-1.1 ± 3.2	-0.9 ± 3.3
SBP, mmHg (SD)	132.0 ± 16.4	141.9 ± 18.2	140.8 ± 18.2	142.3 ± 19.3	147.8 ± 19.0	146.3 ± 18.9
Male (%)	96,536 (40.3)	4,184 (52.0)	9,596 (59.3)	2,622 (52.8)	21,409 (46.7)	8,040 (57.1)
Race (%)						
Non-white	12,054 (5.0)	979 (12.2)	745 (4.6)	183 (3.7)	2,565 (5.6)	919 (6.5)
white	227,737 (95.0)	7,061 (87.8)	15,440 (95.4)	4,782 (96.3)	43,318 (94.4)	13,171 (93.5)
Qualifications (%)						
without college	118,995 (49.6)	3,987 (49.6)	7,811 (48.3)	2,355 (47.4)	22,646 (49.4)	6,758 (48.0)
with college	90,182 (37.6)	1,751 (21.8)	4,362 (27.0)	1,537 (31.0)	11,894 (25.9)	3,304 (23.4)
Alcohol consumption (%)						
Daily or almost daily	46,410 (19.4)	1,144 (14.2)	3,366 (20.8)	1,166 (23.5)	9,631 (21.0)	2,835 (20.1)
Three or four times a week	57,094 (23.8)	1,319 (16.4)	3,348 (20.7)	1,000 (20.1)	9,500 (20.7)	2,706 (19.2)
Once or twice a week	64,477 (26.9)	1,958 (24.4)	4,102 (25.3)	1,222 (24.6)	11,295 (24.6)	3,430 (24.3)
One to three times a month	28,234 (11.8)	1,029 (12.8)	1,737 (10.7)	477 (9.6)	5,011 (10.9)	1,489 (10.6)
Special occasions only	25,900 (10.8)	1,420 (17.7)	2,088 (12.9)	625 (12.6)	6,022 (13.1)	2,012 (14.3)
Never	17,511 (7.3)	1,160 (14.4)	1,517 (9.4)	468 (9.4)	4,345 (9.5)	2,835 (20.1)
Household income (%)						
<£31,000	84,244 (35.1)	4,028 (50.1)	7,479 (46.2)	2,421 (48.8)	21,123 (46.0)	6,942 (49.3)
≥ £31,000	126,187 (52.6)	2,524 (31.4)	6,118 (37.8)	1,719 (34.6)	16,738 (36.5)	4,577 (32.5)
Physical activity (%)						
low	34,474 (14.4)	1,499 (18.6)	2,426 (15.0)	694 (14.0)	6,908 (15.1)	2,198 (15.6)
moderate	80,062 (33.4)	2,314 (28.8)	4,953 (30.6)	1,481 (29.8)	14,065 (30.7)	4,156 (29.5)
high	81,616 (34.0)	2,077 (25.8)	5,242 (32.4)	1,693 (34.1)	13,694 (29.8)	4,221 (30.0)
Health diet score (%)						
0	1,445 (0.6)	79 (1.0)	125 (0.8)	36 (0.7)	336 (0.7)	124 (0.9)
1	12,428 (5.2)	583 (7.3)	958 (5.9)	262 (5.3)	2,405 (5.2)	870 (6.2)
2	39,125 (16.3)	1,529 (19.0)	2,933 (18.1)	795 (16.0)	7,804 (17.0)	2,564 (18.2)
3	67,721 (28.2)	2,327 (28.9)	4,574 (28.3)	1,464 (29.5)	13,202 (28.8)	4,024 (28.6)
4	60,243 (25.1)	1,705 (21.2)	3,765 (23.3)	1,228 (24.7)	11,227 (24.5)	3,250 (23.1)
5	24,438 (10.2)	523 (6.5)	1,384 (8.6)	436 (8.8)	4,057 (8.8)	1,070 (7.6)

Note: BMI: body mass index, SBP: systolic blood pressure, T2D: type II diabetes, CHD: coronary heart diseases, FCMD: first cardiometabolic disease, CMM: cardiometabolic multimorbidity, sd: standard deviation

Among all analyzed participants, during a median follow-up of 13.2 years, 59,193 participants developed a CMD, 8,040 participants occurred T2D, 16,185 suffered from CHD, 4,965 had stroke, and 45,883 had hypertension (Figure S1). Among participants with FCMD, 14,090 further developed CMM. A total of 16,487 deaths were reported during the follow-up. The remaining 239,791 participants without any CMDs were treated as controls. In comparison to participants without any CMDs, those with one CMD or CMM trended to be older, males, have higher BMI, and SBP. Furthermore, they have lower education levels, household income, physical activity level, and health diet scores, or be active smokers and drinkers and have an older age at quitting smoking.

### Association of smoking status with FCMD, CMM and all-cause death

After adjusting for available covariates and using never smokers as the reference, we discovered that current smokers were significantly associated with a higher risk of developing FCMD, CMM, and all-cause death (pattern A) (Table 2). Meanwhile, compared to current smokers, we observed a significantly reduced risk at different transition stages after smoking cessation. Specifically, former smokers not only had a reduced risk of transition from baseline to FCMD (HR [95% CI] = 1.59 [1.55 ∼ 1.63] vs. 1.18 [1.16 ∼ 1.21], *P* = 1.48 × 10^− 118^; here, the effect modification was tested via the *Z*-statistic [43], and the *P* value was obtained by looking up the *Z*-score on a standard normal distribution), but also had a lower risk of transition from FCMD to CMM (1.40 [1.33 ∼ 1.47] vs. 1.09 [1.05 ∼ 1.14], *P* = 1.50 × 10^− 18^) (Table 2). For the transition from other stages to death, quitting smoking was also significantly related to a smaller risk from baseline (2.87 [2.72 ∼ 3.03] vs. 1.38 [1.32 ∼ 1.45], *P* < 0.001), FCMD (2.16 [1.98 ∼ 2.35] vs. 1.25 [1.16 ∼ 1.34], *P* = 1.18 × 10^− 46^), and CMM (2.02 [1.79 ∼ 2.28] vs. 1.22 [1.09 ∼ 1.35], *P* = 3.93 × 10^− 17^) (Table 2). On average, smoking cessation could reduce the risk attributed to cigarette smoking by approximately 76.5% across all transition stages in pattern A.

**Table 2:** Association of smoking status with FCMD, CMM and all-cause death

Association	smoking status	All participants		Pack-years (< 20)		Pack-years (≥ 20)	
		HR (95% CI)	*P*	HR (95% CI)	*P*	HR (95% CI)	*P*
Baseline → FCMD	Never	1 [Reference]	-	1 [Reference]	-	1 [Reference]	-
	Former	1.18 (1.16 ∼ 1.21)	6.74 × 10^− 64^	1.09 (1.06 ∼ 1.12)	2.05 × 10^− 12^	1.32 (1.28 ∼ 1.35)	4.61 × 10^− 93^
	Current	1.59 (1.55 ∼ 1.63)	3.87 × 10^− 261^	1.37 (1.31 ∼ 1.43)	5.58 × 10^− 47^	1.74 (1.69 ∼ 1.79)	1.92 × 10^− 268^
FCMD → CMM	Never	1 [Reference]	-	1 [Reference]	-	1 [Reference]	-
	Former	1.09 (1.05 ∼ 1.14)	7.09 × 10^− 6^	1.04 (0.99 ∼ 1.10)	0.082	1.15 (1.09 ∼ 1.20)	7.53 × 10^− 8^
	Current	1.40 (1.33 ∼ 1.47)	3.36 × 10^− 41^	1.31 (1.21 ∼ 1.43)	3.06 × 10^− 11^	1.45 (1.37 ∼ 1.53)	2.54 × 10^− 38^
Baseline → Death	Never	1 [Reference]	-	1 [Reference]	-	1 [Reference]	-
	Former	1.38 (1.32 ∼ 1.45)	8.36 × 10^− 43^	1.14 (1.07 ∼ 1.21)	2.05 × 10^− 5^	1.82 (1.71 ∼ 1.93)	7.99 × 10^− 84^
	Current	2.87 (2.72 ∼ 3.03)	< 0.001	2.19 (2.00 ∼ 2.39)	7.12 × 10^− 68^	3.37 (3.17 ∼ 3.58)	< 0.001
FCMD → Death	Never	1 [Reference]	-	1 [Reference]	-	1 [Reference]	-
	Former	1.25 (1.16 ∼ 1.34)	2.29 × 10^− 9^	1.07 (0.98 ∼ 1.18)	0.147	1.46 (1.33 ∼ 1.59)	1.65 × 10^− 16^
	Current	2.16 (1.98 ∼ 2.35)	3.03 × 10^− 69^	1.74 (1.50 ∼ 2.02)	4.04 × 10^− 13^	2.36 (2.15 ∼ 2.60)	6.72 × 10^− 70^
CMM → Death	Never	1 [Reference]	-	1 [Reference]	-	1 [Reference]	-
	Former	1.22 (1.09 ∼ 1.35)	3.87 × 10^− 4^	1.07 (0.93 ∼ 1.23)	0.366	1.39 (1.22 ∼ 1.58)	4.90 × 10^− 7^
	Current	2.02 (1.79 ∼ 2.28)	1.02 × 10^− 29^	1.69 (1.36 ∼ 2.10)	1.75 × 10^− 6^	2.12 (1.85 ∼ 2.42)	1.59 × 10^− 28^

Note: Never: never smokers, Former: former smokers, Current: current smokers, FCMD: first cardiometabolic disease, CMM: cardiometabolic multimorbidity

Furthermore, similar results were observed in the low and high pack-years group, and the high pack-years group exhibited a greater risk of transition from baseline to FCMD, CMM and all-cause death (Table S3). On average, current smokers and former smokers in the high pack-years group had a higher risk of transition than those in the low pack-years group, respectively.

### Association of cigarette smoking with FCMD, CMM and all-cause death

#### Association of age at smoking initiation with FCMD, CMM and all-cause death

Table 3 showed the association between age at smoking initiation and the risk for FCMD, CMM and all-cause death. Compared to never smokers, the risk of transition from baseline to FCMD, from FCMD to CMM, and from baseline and FCMD to all-cause death increased with earlier age at smoking initiation. The HR (95% CI) of transition from baseline to FCMD was 1.70 (1.64 ∼ 1.75), from FCMD to CMM was 1.43 (1.35 ∼ 1.52), from baseline, FCMD and CMM to death was 3.14 (2.94 ∼ 3.36), 2.26 (2.03 ∼ 2.51) or 2.01 (1.73 ∼ 2.34) for those started smoking < 18 years. Moreover, the analyses grouped by pack-years revealed similar findings, highlighting a notably elevated risk of transition from baseline to FCMD, CMM, and all-cause mortality among the high pack-years group (Table 3).

**Table 3:** Association of smoking initiation with FCMD, CMM and all-cause death

Association	smoking status	All participants		Pack-years (< 20)		Pack-years (≥ 20)	
		HR (95% CI)	*P*	HR (95% CI)	*P*	HR (95% CI)	*P*
Baseline → FCMD	Never	1 [Reference]	-	1 [Reference]	-	1 [Reference]	-
	≥ 18	1.47 (1.41 ∼ 1.52)	3.36 × 10^− 83^	1.32 (1.24 ∼ 1.39)	3.75 × 10^− 21^	1.61 (1.63 ∼ 1.70)	4.73 × 10^− 77^
	< 18	1.70 (1.64 ∼ 1.75)	6.96 × 10^− 218^	1.45 (1.36 ∼ 1.54)	1.83 × 10^− 30^	1.80 (1.83 ∼ 1.87)	7.00 × 10^− 215^
FCMD → CMM	Never	1 [Reference]	-	1 [Reference]	-	1 [Reference]	-
	≥ 18	1.36 (1.27 ∼ 1.46)	1.32 × 10^− 17^	1.31 (1.18 ∼ 1.46)	9.32 × 10^− 7^	1.41 (1.43 ∼ 1.54)	8.27 × 10^− 14^
	< 18	1.43 (1.35 ∼ 1.52)	1.59 × 10^− 31^	1.32 (1.17 ∼ 1.48)	5.20 × 10^− 6^	1.47 (1.43 ∼ 1.57)	4.10 × 10^− 30^
Baseline → Death	Never	1 [Reference]	-	1 [Reference]	-	1 [Reference]	-
	≥ 18	2.55 (2.36 ∼ 2.76)	2.97 × 10^− 125^	2.06 (1.83 ∼ 2.31)	3.85 × 10^− 33^	3.12 (3.13 ∼ 3.44)	1.97 × 10^− 119^
	< 18	3.14 (2.94 ∼ 3.36)	2.55 × 10^− 256^	2.29 (2.02 ∼ 2.61)	1.76 × 10^− 36^	3.56 (3.53 ∼ 3.82)	9.22 × 10^− 261^
FCMD → Death	Never	1 [Reference]	-	1 [Reference]	-	1 [Reference]	-
	≥ 18	2.01 (1.78 ∼ 2.27)	5.88 × 10^− 29^	1.56 (1.27 ∼ 1.91)	2.54 × 10^− 5^	2.35 (2.33 ∼ 2.72)	3.30 × 10^− 31^
	< 18	2.26 (2.03 ∼ 2.51)	6.81 × 10^− 53^	1.98 (1.61 ∼ 2.44)	1.10 × 10^− 10^	2.37 (2.33 ∼ 2.66)	1.29 × 10^− 50^
CMM → Death	Never	1 [Reference]	-	1 [Reference]	-	1 [Reference]	-
	≥ 18	1.97 (1.67 ∼ 2.33)	8.49 × 10^− 16^	1.75 (1.33 ∼ 2.30)	7.00 × 10^− 5^	2.08 (2.03 ∼ 2.52)	8.84 × 10^− 14^
	< 18	2.01 (1.73 ∼ 2.34)	3.54 × 10^− 20^	1.41 (1.00 ∼ 1.97)	0.048	2.15 (2.13 ∼ 2.52)	1.41 × 10^− 21^

Note: Never: never smokers, FCMD: first cardiometabolic disease, CMM: cardiometabolic multimorbidity

#### Association of pack-years of smoking with FCMD, CMM and all-cause death

Relative to never smoking, increased smoking also significantly elevated the risk of FCMD, CMM, and all-cause death (Table 4). Taking the transition from baseline to FCMD as an example, the HR (95% CI) for individuals who smoked more than 30 pack-years was 1.53 (1.49 ∼ 1.57); however, the HR was only 1.06 (1.03 ∼ 1.09) for those who smoked up to 10 pack-years. Compared to individuals who smoked more than 30 pack-years, those who smoked up to 10 pack-years could have an approximately 90.2% lower risk attributed to cigarette smoking. In addition, the risk of FCMD, CMM and all-cause death increased by an average of 11.0% for every 10 units of pack-years of smoking (Table 4).

**Table 4:** Association of pack-years of smoking with the risk of FCMD, CMM and all-cause death

Association	Pack-years of smoking	All participants	
		HR (95% CI)	*P*
Baseline → FCMD	None	1 [Reference]	-
	Up to 10	1.06 (1.03 ∼ 1.09)	2.82 × 10^− 4^
	Between 11 and 20	1.19 (1.16 ∼ 1.22)	2.67 × 10^− 32^
	Between 21 and 30	1.35 (1.31 ∼ 1.39)	1.24 × 10–^79^
	More than 30	1.53 (1.49 ∼ 1.57)	1.40 × 10^− 219^
	HR per 10 units	1.08 (1.08 ∼ 1.08)	1.42 × 10^− 60^
FCMD → CMM	None	1 [Reference]	-
	Up to 10	1.04 (0.97 ∼ 1.11)	0.254
	Between 11 and 20	1.14 (1.07 ∼ 1.20)	8.83 × 10^− 6^
	Between 21 and 30	1.19 (1.12 ∼ 1.26)	1.88 × 10^− 8^
	More than 30	1.29 (1.23 ∼ 1.35)	3.12 × 10^− 26^
	HR per 10 units	1.05 (1.05 ∼ 1.05)	4.37 × 10^− 9^
Baseline → Death	None	1 [Reference]	-
	Up to 10	1.09 (1.01 ∼ 1.18)	0.022
	Between 11 and 20	1.40 (1.31 ∼ 1.50)	8.85 × 10^− 23^
	Between 21 and 30	1.85 (1.72 ∼ 1.98)	1.46 × 10^− 66^
	More than 30	2.72 (2.57 ∼ 2.87)	1.88 × 10^− 281^
	HR per 10 units	1.18 (1.18 ∼ 1.18)	4.72 × 10^− 64^
FCMD → Death	None	1 [Reference]	-
	Up to 10	1.03 (0.91 ∼ 1.17)	0.622
	Between 11 and 20	1.24 (1.11 ∼ 1.38)	1.02 × 10^− 4^
	Between 21 and 30	1.37 (1.23 ∼ 1.53)	2.75 × 10^− 8^
	More than 30	1.98 (1.83 ∼ 2.15)	2.17 × 10^− 61^
	HR per 10 units	1.13 (1.13 ∼ 1.13)	1.39 × 10^− 20^
CMM → Death	None	1 [Reference]	-
	Up to 10	1.12 (0.93 ∼ 1.36)	0.228
	Between 11 and 20	1.10 (0.93 ∼ 1.29)	0.270
	Between 21 and 30	1.43 (1.23 ∼ 1.68)	5.62 × 10^− 6^
	More than 30	1.78 (1.59 ∼ 1.99)	3.48 × 10^− 23^
	HR per 10 units	1.11 (1.11 ∼ 1.11)	7.60 × 10^− 9^

Note: FCMD: first cardiometabolic disease, CMM: cardiometabolic multimorbidity

### Association of age at smoking cessation with FCMD, CMM, and all-cause death

**Fig. 2:**
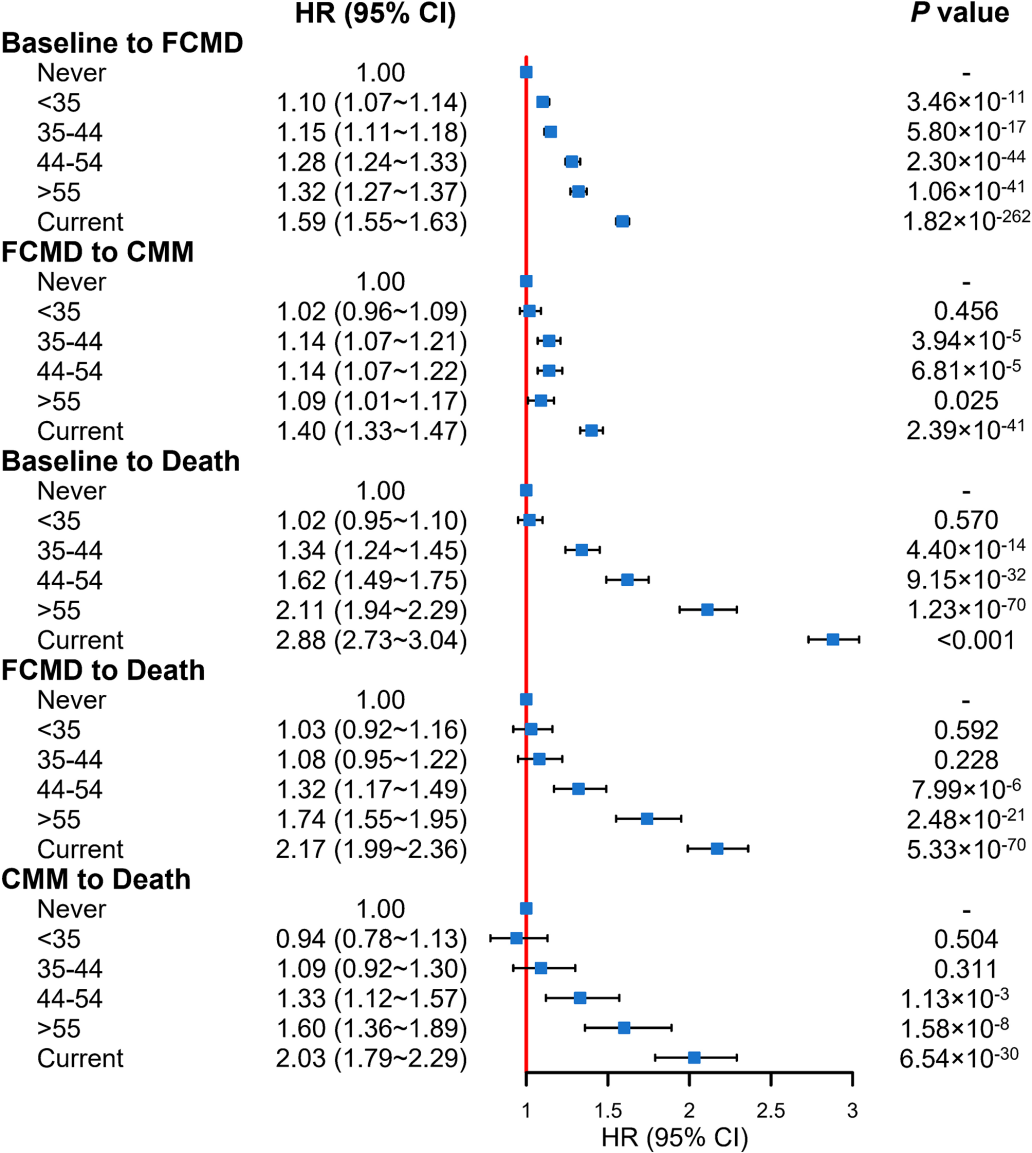
Forest diagram representing the association of cigarette smoking, smoking cessation with FCMD, CMM, and all-cause death by various ages at quitting smoking, compared to never and current smokers in pattern A. Never: never smokers, Current: current smokers, FCMD: first cardiometabolic disease, CMM: cardiometabolic multimorbidity

The above associations remained substantial when using age at quitting smoking as a proxy for former smokers in pattern A (Fig. 2). Compared to current smokers, we discovered that all the risks of transition from baseline to FCMD, from FCMD to CMM, and from baseline and FCMD to all-cause death in general reduced progressively when the age at quitting became younger. In contrast, current smokers had the higher risk at each transition. Taking the transition from baseline to FCMD as an example, the HR (95% CI) for current smokers was 1.59 (1.55 ∼ 1.63); however, the HR was only 1.10 (1.07 ∼ 1.14) for quitting smoking before age 35 years, 1.15 (1.11 ∼ 1.18) for quitting smoking at ages 35 to 44 years, 1.28 (1.24 ∼ 1.33) for quitting smoking at ages 45 to 54 years, and 1.32 (1.27 ∼ 1.37) for quitting smoking at 55 years or more. Quitting smoking at age < 35 years was linked with a reduction of about 98.8% of the risk attributed to cigarette smoking, even quitting smoking at age ≥ 55 years was also attributed to an approximately 48.5% decrease in risk in pattern A.

Furthermore, we grouped participants by pack-years, and analyzed those who quit smoking before and after age 45 in two groups. We also found similar results to those described above, and the high pack-years group showed a higher risk in pattern A (Table S3).

### Association of age at smoking initiation and smoking cessation with individual FCMD

In the analysis of individual FCMDs (pattern B), we discovered similar association patterns for age at smoking initiation except for the transition from T2D to CMM and death, CHD to CMM, and CMM to death (Table S4). Taking the transition from hypertension to CMM as an example, the HR (95% CI) for participants starting smoking < 18 years was 1.61 (1.48 ∼ 1.74), whereas the HR (95% CI) was 1.46 (1.33 ∼ 1.61) for those starting smoking < 18 years.

We also observed similar association patterns for age at smoking cessation except for the transition from T2D to CMM (Table S5). Taking the transition from hypertension to CMM as an example, the HR (95% CI) for current smokers was 1.46 (1.39 ∼ 1.54), whereas the HR (95% CI) was 1.14 (1.08 ∼ 1.21) for quitting smoking age at 45 years or more, and only 1.07 (1.02 ∼ 1.13) for quitting smoking before age 45 years. Quitting smoking at age before 45 years could reduce the risk related to cigarette smoking by about 85.1% when transiting from baseline to hypertension, CMM and Death.

### Subgroup and sensitive analyses

In the subgroup analyses conducted at different levels of a given covariate, we observed that the risk of transitions related to current smoking was generally similar, with current smokers having the higher risk at all the five transition stages in pattern A and at all the fourteen transition stages in pattern B (Figures S2-S8). In contrast, former smokers were connected with a decreased risk of transition compared to current smokers regardless of the level of covariates (Figures S2-S8). Particularly, the associations of cigarette smoking and smoking cessation with CMM were generally consistent between males and females. Interestingly, however, the risk reduction from smoking cessation appeared to be more pronounced for females compared to males (on average 79.0% in females vs. 66.8% in males) (Table S6 and Figure S4).

In the sensitivity analyses with only white participants, almost all the results remained relatively robust when smokers who quit smoking due to illness/disease and those who stopped smoking due to physician recommendation were excluded (Figure S9), and nearly all the associations were still significant when participants with CMD events occurring within the first two years of follow-up, and participants with smoking cessation within two years before and after recruitment were removed (Figure S10).

### Attributable risk and population attributable risk of FCMD, CMM and all-cause death

The AF% and PAF% results were shown in Table S7. Compared to never smokers, among ever smokers, an estimated 22.3% of cases from baseline to FCMD, 42.8% of cases from FCMD to CMM, 33.9% of deaths from baseline, 16.8% of deaths from FCMD, and 32.8% of deaths from CMM were attributable to cigarette smoking. Among the general population, if people quit smoking, the disease or death transition states would not occur for 8.9% of the population from baseline to FCMD, 20.3% from FCMD to CMM, 14.9% from baseline to death, 6.4% from FCMD to death from any cause, and 14.2% from CMM to death from any cause.

In particular, there seemed to be only a very small difference in AF% or PAF% between male and female participants, but we observed generally higher AF% and PAF% in males, which may be due to the effects of smoking and differences in smoking prevalence. For example, about 26.4% of cases from baseline to CHD were attributable to cigarette smoking among females, but this estimate was 36.2% among males. Among the general population, approximately 16.1% of deaths from stroke were attributable to cigarette smoking among females, whereas this estimate was 19.6% among males. Overall, our findings indicate that cigarette smoking is associated with a high risk for all transition stages of CMM in both males and females.

## Discussion

### Summary of our study

In this study, we have leveraged the large prospective UK Biobank cohort to examine the association of cigarette smoking and smoking cessation with the trajectory of CMM, from onset, progression, to death. We demonstrated that cigarette smoking substantially increased the risk of all transition stages, especially when the age of initiation was before 18 years; however, smoking cessation could reduce even eliminate such a risk, and could still play a substantial role in decreasing the risk of CMD-specific transition. When considering age at smoking initiation and smoking cessation, we discovered that the earlier people quit smoking, the more substantial the risk reduction. Importantly, we revealed that the risk of disease or condition stage transition for former smokers would reduce to almost the same level as for never smokers when the age of smoking cessation was less than 35 years.

### Comparison to previous studies

The effects of cigarette smoking and smoking cessation on certain transitions in CMM were largely in agreement with those observed in prior studies [7, 13, 14], which however only reported the association of cigarette smoking and smoking cessation with one single disease stage of CMM, and failed to assess the effects of cigarette smoking and cessation on different transition stages throughout the CMM process. For example, many studies showed an association between smoking cessation and a reduced incidence of individual CMDs [7, 13, 14], consistent with the decreased risk of transition from baseline to FCMD uncovered in our study. In addition, several studies reported an association between cigarette smoking and an increase in incident FCMD and all-cause mortality [30, 44–46]. Previous studies also indicated an increased risk of CMM due to cigarette smoking in patients with hypertension [42]. In the present work, the multi-state model was applied to account for the transition through different disease stages, which is commonly employed to explore the role of risk factors in the progression of CMM, including lifestyle, clinical and behavioral factors [15, 41]. Therefore, this model is well-suited for our research application.

After considering age at quitting smoking used as a proxy for former smokers in the multi-stage model, the results showed the dose-response relationships between age at quitting smoking and different transition stages of the CMM, which is also consistent with the findings in previous studies that examined only a single stage of CMM [12, 30]. Specifically, the risks of from baseline to FCMD, from FCMD to CMM, from baseline to death, from FCMD to death, and from CMM to death all reduced as the age at quitting smoking became younger. Particularly, we discovered that the risk of cigarette smoking on CMM was decreased almost to the level of never smokers when stopping smoking before the age of 35 years.

Finally, again with the help of the multi-stage model, we demonstrated that the harms of cigarette smoking and the benefits of smoking cessation persisted after the diagnosis of FCMD and that smoking cessation could reduce the risk of FCMD to CMM or further to death. Similar results were consistently observed for the four specific CMDs.

### Public health implications of our findings

Our findings have significant public health implications. First, as the earlier smoking initiation, the greater the risk for CMM at different transition stages. Therefore, reducing the accessibility of cigarettes is one of the most effective ways to prevent adolescents from smoking [47].

Second, smoking cessation plays a role in reducing the risk of CMM at different transition stages, and a dose-response relationship between age at quitting smoking and CMM was found, suggesting that earlier smoking cessation should be specifically emphasized when designing smoking-relevant health protection strategies.

The global phenomenon of population ageing is consistently related to an increased burden of chronic diseases (especially CMDs) and an increase in disability-adjusted life years [48], and smoking cessation (especially at a young age) is associated with a substantial reduction in the risk of CMM, which has an important role in extending healthy life expectancy in old age and reducing the socioeconomic burden of population ageing.

Our study also helps us identify the profound benefits of smoking cessation; by utilizing the multi-stage model, we found that the effects of smoking cessation persisted even after the diagnosis of CMD. Therefore, active smoking cessation counseling is needed for patients with CMD to reduce the risk of transition from FCMD to CMM and/or death.

### Strengths of this study

Our study has several strengths. First, the major strength is the use of the multi-state model rather than a conventional Cox model, which allows us to assess the associations of cigarette smoking and smoking cessation with CMM at different transition stages [28, 39, 41]. Second, the large sample size of the UK Biobank cohort provides substantial power, allowing us to further investigate all transitions of specific individual CMDs, and obtain a deeper insight into the association between smoking cessation and CMM [49, 50]. Third, the UK Biobank included the detailed information of smoking behaviors such as smoking status, age at smoking initiation, age at smoking cessation and pack-years of smoking. Finally, the prospective analysis guarantees a clear temporal sequence between cigarette smoking, smoking cessation and CMM during distinct transition stages, thus reducing potential inverse confounding [41].

### Limitations of this study

Some limitations of this study should be mentioned. First, the information on cigarette smoking and smoking cessation was self-reported and collected retrospectively, which may be subject to recall bias and social desirability bias [42, 51]. Due to social desirability bias, people tend to over-report socially desirable behaviors and underreport undesirable behaviors [52]. This is likely to cause some participants who were current smokers to be wrongly categorized as former or never smokers. The effect of quitting smoking may also be underestimated.

Second, we used information on cigarette smoking, smoking cessation, and covariates at baseline, which likely changed over time during the follow-up [21]. Some participants possibly modified their lifestyles after the diagnosis of FCMD, such as quitting smoking, increasing exercise, and eating a healthier diet. These potential improvements in health behaviors after FCMD diagnosis were likely to result in an underestimation of observed associations [42]. However, it has been shown that, in the absence of intervention, most people were unlikely to change these behaviors after a newly diagnosed condition [53, 54].

Finally, the UK Biobank cohort includes mainly white people from developed regions [55], which may affect the generalization of results to other populations especially to non-white people in low/middle-income countries or regions, which likely present different disease spectrums, lifestyles, and behavioral habits [48, 56, 57].

## Conclusions

Cigarette smoking was associated with a higher risk of CMM across all transitions; however, smoking cessation, especially before the age of 35, was associated with a significant decrease in CMM risk attributed to cigarette smoking.

### Electronic supplementary material

Below is the link to the electronic supplementary material.

Supplementary Material 1

## Data Availability

This study used the UK Biobank resource with the application ID 88159. Researchers can access to the UK Biobank dataset by applying to the UK Biobank official website (https://www.ukbiobank.ac.uk/). All data generated or analysed during this study are included in this published article and its supplementary information files.

## Abbreviations

CMD: Cardiometabolic disease
FCMD: First cardiometabolic disease
CMM: Cardiometabolic multimorbidity
CHD: Coronary heart disease
T2D: Type II diabetes
CI: Confidence interval
HR: Hazard ratio
SD: Standard deviation
BMI: Body mass index
IPAQ: International physical activity questionnaire
SBP: Systolic blood pressure
