# Supplementary material for: Association of cigarette smoking, smoking cessation with the risk of cardiometabolic multimorbidity in the UK Biobank
Source: BMC Public Health. 2024 Jul 16;24:1910. doi: 10.1186/s12889-024-19457-y (PMC11253396; doi:10.1186/s12889-024-19457-y)
Supplement: Supplementary file 1 — Supplementary Material 1 [file 12889_2024_19457_MOESM1_ESM.docx]

**Supplementary File**

**Treatment of covariates**

A wide range of sociodemographic characteristics and lifestyle factors were considered as covariates to adjust for any potential confounding. Among these covariates, income was classified into two levels with a cutoff of £31,000, which was close to the UK median household income in October 2009 (£27,530) [[1](#_ENREF_1)].

Physical activity was assessed by the mean of the International Physical Activity Questionnaire (IPAQ) short form, according to which physical activity was classified into three groups: low (<600 min/week), moderate (between 600-3000 min/week), and high (>3000 min/week) [[2](#_ENREF_2)].

Healthy diet score was calculated in terms of daily dietary factors ranging from 0 to 5: one point was scored when each of the following favorable conditions was met: (1) vegetable intake ≥4 tablespoons/day; (2) fruit intake ≥3 pieces/day; (3) fish intake ≥2 times/week; (4) unprocessed red meat intake ≤2 times/week; (5) and processed meat intake ≤2 times/week [[3](#_ENREF_3)].

Systolic blood pressure (SBP) was measured by trained nurses using standardized procedures during the initial assessment center visit. SBP was calculated as the average of blood pressure readings when both automated and manual measurements could be obtained. Considering that medication for blood pressure can lead to the decrease in both SBP, and thus biased the relationship, we added 15 mmHg for SBP for participants who received the special medication for blood pressure (fields 6153 and 6177) [[4](#_ENREF_4), [5](#_ENREF_5)].

Table S1. Definition of the four focused cardiometabolic diseases in the UK Biobank.

| Cardiometabolic diseases | Self-reported | ICD-9 | ICD-10 | OPCS-4 |
| --- | --- | --- | --- | --- |
| Type II diabetes | 2443 (1), 2976, 6153 (3), 6177 (3), 20002 (1223), 20008, 20009 | - | 41270 (E11), 41280 | - |
| Coronary heart diseases | 6150 (1, 2), 3894, 3627, 20004 (1070, 1095, 1523), 20002 (1074, 1075), 20008, 20009, 20010, 20011 | 41271 (410-414), 41281 | 41270 (I20-I25, Z951, Z955), 41280 | 41272 (K40-K46, K49, K50, K75), 41282 |
| Stroke | 6150 (3), 4056, 20002 (1081, 1491, 1583, 1086), 20008, 20009, 20010, 20011 | 41271 (3361, 3623, 430, 431, 4329, 4330, 4331, 4332,4333, 4338, 4339, 434, 436), 41281 | 41270 (I60, I61, I629, I63, I64, I678, I690, I693, G951, H341, H342, S066), 41280 | 41272 (A052-A054, L351, L353, L343), 41282 |
| Hypertension | 2966, 6150 (4), 6153 (2), 6177 (2), 20002 (1065, 1072), 20008, 20009 | 41271 (401-405) 41281 | 41270 (I10-I13, I15, O10), 41280, | - |

Note: ICD-9: International Classification of Disease version 9; ICD-10: International Classification of Disease version 10; OPCS-4: Office of Population Censuses and Surveys Classification of Interventions and Procedures, version 4.

# Table S2. Sources of information for cigarette smoking and smoking cessation in the UK Biobank

| **S**ources | Smoking status | Amount smoked | Age started smoking | Age stopped smoking |
| --- | --- | --- | --- | --- |
| Fields | 1239, 1249, 20116, 22506 | 2644, 2887, 3456, 6183, 10895 | 2867, 3436 | 2897, 6194, 22507 |

# Table S3. Association of age at smoking cessation with FCMD, CMM and all-cause death by pack-year subgroups

| Association | Pack-years (<20) | | |  | Pack-years (<20) | | |
| --- | --- | --- | --- | --- | --- | --- | --- |
|  | smoking status | HR (95%CI) | *P* |  | smoking status | HR (95%CI) | *P* |
| **Baseline→ FCMD** | Never | 1 [Reference] | - |  | Never | 1 [Reference] | - |
|  | <45 | 1.07 (1.04~1.10) | 1.70×10^-7^ |  | <45 | 1.26 (1.21~1.32) | 1.33×10^-30^ |
|  | ≥45 | 1.17 (1.12~1.23) | 2.56×10^-10^ |  | ≥45 | 1.35 (1.31~1.40) | 4.01×10^-77^ |
|  | Current | 1.37 (1.31~1.43) | 6.34×10^-47^ |  | Current | 1.74 (1.69~1.79) | 1.32×10^-268^ |
| **FCMD → CMM** | Never | 1 [Reference] | - |  | Never | 1 [Reference] | - |
|  | <45 | 1.04 (0.99~1.10) | 0.115 |  | <45 | 1.15 (1.07~1.24) | 2.06×10^-4^ |
|  | ≥45 | 1.05 (0.95~1.15) | 0.352 |  | ≥45 | 1.14 (1.08~1.21) | 9.59×10^-6^ |
|  | Current | 1.31 (1.21~1.43) | 3.06×10^-11^ |  | Current | 1.45 (1.37~1.53) | 2.58E-38 |
| **Baseline→ Death** | Never | 1 [Reference] | - |  | Never | 1 [Reference] | - |
|  | <45 | 1.08 (1.02~1.16) | 0.015 |  | <45 | 1.43 (1.30~1.58) | 1.21×10^-12^ |
|  | ≥45 | 1.37 (1.23~1.54) | 3.53×10^-8^ |  | ≥45 | 2.06 (1.92~2.20) | 6.82×10^-93^ |
|  | Current | 2.19 (2.00~2.39) | 8.23×10^-68^ |  | Current | 3.37 (3.17~3.58) | <0.001 |
| **FCMD → Death** | Never | 1 [Reference] | - |  | Never | 1 [Reference] | - |
|  | <45 | 1.00 (0.90~1.11) | 0.993 |  | <45 | 1.20 (1.03~1.39) | 0.019 |
|  | ≥45 | 1.33 (1.13~1.57) | 4.83×10^-4^ |  | ≥45 | 1.59 (1.44~1.76) | 1.39×10^-19^ |
|  | Current | 1.74 (1.50~2.02) | 4.05×10^-13^ |  | Current | 2.37 (2.15~2.61) | 3.49×10^-70^ |
| **CMM → Death** | Never | 1 [Reference] | - |  | Never | 1 [Reference] | - |
|  | <45 | 1.05 (0.90~1.23) | 0.552 |  | <45 | 0.99 (0.79~1.23) | 0.923 |
|  | ≥45 | 1.13 (0.88~1.45) | 0.346 |  | ≥45 | 1.59 (1.38~1.83) | 8.06×10^-11^ |
|  | Current | 1.69 (1.36~2.09) | 1.76×10^-6^ |  | Current | 2.12 (1.86~2.42) | 1.40×10^-28^ |

Note: Never: never smokers, Current: current smokers, FCMD: first cardiometabolic disease, CMM: cardiometabolic multimorbidity.

# Table S4. Association of age at smoking initiation with FCMD in participants without any CMDs, and subsequent all-cause death in pattern B

| Association | | without any CMD | |  | with FCMD | | | |  | with FCMD | | | |
| --- | --- | --- | --- | --- | --- | --- | --- | --- | --- | --- | --- | --- | --- |
|  |  | HR (95%CI) | *P* |  | Association | | HR (95%CI) | *P* |  | Association | | HR (95%CI) | *P* |
| **Baseline → T2D** | | | |  | **T2D → CMM** | | | |  | **T2D → Death** | | | |
|  | Never | 1 [Reference] | - |  |  | Never | 1 [Reference] | - |  |  | Never | 1 [Reference] | - |
|  | ≥18 | 1.75 (1.56~1.96) | 1.92×10^-21^ |  |  | ≥18 | 1.41 (1.18~1.68) | 1.92×10^-4^ |  |  | ≥18 | 2.02 (1.54~2.64) | 3.05×10^-7^ |
|  | <18 | 2.39 (2.19~2.62) | 6.16×10^-79^ |  |  | <18 | 1.25 (1.08~1.45) | 0.003 |  |  | <18 | 1.78 (1.41~2.26) | 1.77×10^-6^ |
| **Baseline→ CHD** | | | |  | **CHD→ CMM** | | | |  | **CHD→ Death** | | | |
|  | Never | 1 [Reference] | - |  |  | Never | 1 [Reference] | - |  |  | Never | 1 [Reference] | - |
|  | ≥18 | 1.78 (1.65~1.92) | 1.12×10^-47^ |  |  | ≥18 | 1.20 (1.07~1.34) | 0.002 |  |  | ≥18 | 2.43 (2.02~2.92) | 4.60×10^-21^ |
|  | <18 | 2.19 (2.06~2.34) | 1.28×10^-127^ |  |  | <18 | 1.16 (1.05~1.28) | 0.003 |  |  | <18 | 2.59 (2.20~3.04) | 1.12×10^-30^ |
| **Baseline→ stroke** | | | |  | **Stroke → CMM** | | | |  | **Stroke → Death** | | | |
|  | Never | 1 [Reference] | - |  |  | Never | 1 [Reference] | - |  |  | Never | 1 [Reference] | - |
|  | ≥18 | 2.09 (1.84~2.36) | 3.21×10^-31^ |  |  | ≥18 | 1.14 (0.95~1.37) | 0.168 |  |  | ≥18 | 1.51 (1.23~1.86) | 1.01×10^-4^ |
|  | <18 | 2.10 (1.87~2.35) | 8.60×10^-38^ |  |  | <18 | 1.20 (1.02~1.42) | 0.031 |  |  | <18 | 1.53 (1.25~1.88) | 3.69×10^-5^ |
| **Baseline→ hypertension** | | | |  | **Hypertension → CMM** | | | |  | **Hypertension → Death** | | | |
|  | Never | 1 [Reference] | - |  |  | Never | 1 [Reference] | - |  |  | Never | 1 [Reference] | - |
|  | ≥18 | 1.41 (1.35~1.48) | 3.95×10^-44^ |  |  | ≥18 | 1.46 (1.33~1.61) | 5.50×10^-15^ |  |  | ≥18 | 2.17 (1.91~2.46) | 3.02×10^-33^ |
|  | <18 | 1.59 (1.52~1.66) | 2.63×10^-103^ |  |  | <18 | 1.61 (1.48~1.74) | 1.85×10^-31^ |  |  | <18 | 2.29 (2.05~2.55) | 2.91×10^-48^ |
| **Baseline→ Death** | | | |  | **CMM→ Death** | | | |  |  |  |  |  |
|  | Never | 1 [Reference] | - |  |  | Never | 1 [Reference] | - |  |  |  |  |  |
|  | ≥18 | 2.55 (2.36~2.76) | 2.97×10^-125^ |  |  | ≥18 | 2.14 (1.77~2.58) | 2.28×10^-15^ |  |  |  |  |  |
|  | <18 | 3.14 (2.94~3.36) | 2.55×10^-256^ |  |  | <18 | 1.98 (1.66~2.36) | 2.28×10^-14^ |  |  |  |  |  |

Note: Never: never smokers, T2D: type II diabetes, CHD: coronary heart diseases, CMM: cardiometabolic multimorbidity.

# Table S5. Association of age at smoking cessation with FCMD in participants without any CMDs, and subsequent all-cause death in pattern B

| Association | | without any CMD | |  | with FCMD | | | |  | with FCMD | | | |
| --- | --- | --- | --- | --- | --- | --- | --- | --- | --- | --- | --- | --- | --- |
|  |  | HR (95%CI) | *P* |  | Association | | HR (95%CI) | *P* |  | Association | | HR (95%CI) | *P* |
| **Baseline → T2D** | | | |  | **T2D → CMM** | | | |  | **T2D → Death** | | | |
|  | Never | 1 [Reference] | - |  |  | Never | 1 [Reference] | - |  |  | Never | 1 [Reference] | - |
|  | <45 | 1.20 (1.13~1.28) | 1.53×10^-8^ |  |  | <45 | 1.02 (0.93~1.11) | 0.672 |  |  | <45 | 1.14 (0.94~1.37) | 0.178 |
|  | ≥45 | 1.63 (1.52~1.75) | 4.59×10^-42^ |  |  | ≥45 | 0.98 (0.89~1.07) | 0.637 |  |  | ≥45 | 1.41 (1.18~1.68) | 1.36×10^-4^ |
|  | Current | 2.13 (1.99~2.26) | 3.22×10^-120^ |  |  | Current | 1.21 (1.11~1.32) | 1.48×10^-5^ |  |  | Current | 1.94 (1.64~2.29) | 7.77×10^-15^ |
| **Baseline→ CHD** | | | |  | **CHD→ CMM** | | | |  | **CHD→ Death** | | | |
|  | Never | 1 [Reference] | - |  |  | Never | 1 [Reference] | - |  |  | Never | 1 [Reference] | - |
|  | <45 | 1.18 (1.13~1.23) | 3.74×10^-13^ |  |  | <45 | 1.06 (1.00~1.13) | 0.038 |  |  | <45 | 1.05 (0.91~1.21) | 0.498 |
|  | ≥45 | 1.43 (1.35~1.50) | 1.35×10^-41^ |  |  | ≥45 | 1.05 (0.98~1.12) | 0.164 |  |  | ≥45 | 1.63 (1.42~1.86) | 1.78×10^-12^ |
|  | Current | 2.02 (1.93~2.11) | 5.02×10^-193^ |  |  | Current | 1.13 (1.06~1.21) | 8.41×10^-5^ |  |  | Current | 2.45 (2.17~2.77) | 1.39×10^-46^ |
| **Baseline→ stroke** | | | |  | **Stroke → CMM** | | | |  | **Stroke → Death** | | | |
|  | Never | 1 [Reference] | - |  |  | Never | 1 [Reference] | - |  |  | Never | 1 [Reference] | - |
|  | <45 | 1.09 (1.00~1.18) | 0.044 |  |  | <45 | 1.08 (0.96~1.21) | 0.203 |  |  | <45 | 0.98 (0.84~1.16) | 0.842 |
|  | ≥45 | 1.33 (1.21~1.46) | 1.78×10^-9^ |  |  | ≥45 | 1.07 (0.95~1.22) | 0.268 |  |  | ≥45 | 1.12 (0.95~1.34) | 0.184 |
|  | Current | 2.11 (1.94~2.29) | 2.40×10^-70^ |  |  | Current | 1.13 (1.00~1.27) | 0.044 |  |  | Current | 1.52 (1.31~1.76) | 5.04×10^-8^ |
| **Baseline→** **hypertension** | | | |  | **Hypertension → CMM** | | | |  | **Hypertension →** **Death** | | | |
|  | Never | 1 [Reference] | - |  |  | Never | 1 [Reference] | - |  |  | Never | 1 [Reference] | - |
|  | <45 | 1.14 (1.11~1.17) | 1.29×10^-21^ |  |  | <45 | 1.07 (1.02~1.13) | 0.004 |  |  | <45 | 1.04 (0.95~1.14) | 0.395 |
|  | ≥45 | 1.29 (1.25~1.33) | 4.98×10^-56^ |  |  | ≥45 | 1.14 (1.08~1.21) | 2.04×10^-6^ |  |  | ≥45 | 1.54 (1.41~1.68) | 3.43×10^-22^ |
|  | Current | 1.54 (1.50~1.59) | 2.28×10^-166^ |  |  | Current | 1.46 (1.39~1.54) | 5.39×10^-48^ |  |  | Current | 2.17 (1.99~2.36) | 3.49×10^-72^ |
| **Baseline→ Death** | | | |  | **CMM→ Death** | | | |  |  |  |  |  |
|  | Never | 1 [Reference] | - |  |  | Never | 1 [Reference] | - |  |  |  |  |  |
|  | <45 | 1.16 (1.09~1.22) | 6.62×10^-7^ |  |  | <45 | 1.01 (0.86~1.18) | 0.949 |  |  |  |  |  |
|  | ≥45 | 1.82 (1.71~1.93) | 2.75×10^-81^ |  |  | ≥45 | 1.37 (1.17~1.60) | 7.80×10^-5^ |  |  |  |  |  |
|  | Current | 2.88 (2.73~3.04) | <0.001 |  |  | Current | 2.06 (1.79~2.37) | 1.71×10^-23^ |  |  |  |  |  |

Note: Never: never smokers, Current: current smokers, T2D: type II diabetes, CHD: coronary heart diseases, CMM: cardiometabolic multimorbidity, FCMD: first cardiometabolic disease.

# Table S6. Association of cigarette smoking and smoking cessation with FCMD, CMM, and all-cause death in the subgroup analysis of sex

| Association | smoking status | Female | |  | Male | |
| --- | --- | --- | --- | --- | --- | --- |
|  |  | HR (95%CI) | *P* |  | HR (95%CI) | *P* |
| **Baseline→ FCMD** | Never | 1 [Reference] | - |  | 1 [Reference] | - |
|  | Former | 1.15 (1.13~1.18) | 1.73×10^-30^ |  | 1.14 (1.11~1.17) | 1.09×10^-25^ |
|  | Current | 1.60 (1.54~1.66) | 1.12×10^-139^ |  | 1.45 (1.40~1.49) | 2.06×10^-103^ |
| **FCMD → CMM** | Never | 1 [Reference] | - |  | 1 [Reference] | - |
|  | Former | 1.04 (0.98~1.10) | 0.167 |  | 1.10 (1.05~1.15) | 1.09×10^-4^ |
|  | Current | 1.50 (1.40~1.62) | 4.04×10^-28^ |  | 1.29 (1.22~1.37) | 4.26×10^-17^ |
| **Baseline→ Death** | Never | 1 [Reference] | - |  | 1 [Reference] | - |
|  | Former | 1.30 (1.23~1.38) | 5.60×10^-19^ |  | 1.27 (1.20~1.35) | 4.74×10^-15^ |
|  | Current | 2.59 (2.40~2.80) | 1.35×10^-131^ |  | 2.59 (2.42~2.78) | 4.62×10^-156^ |
| **FCMD → Death** | Never | 1 [Reference] | - |  | 1 [Reference] | - |
|  | Former | 1.08 (0.98~1.19) | 0.131 |  | 1.23 (1.13~1.35) | 6.28×10^-6^ |
|  | Current | 2.16 (1.91~2.45) | 2.21×10^-34^ |  | 1.96 (1.75~2.19) | 1.16×10^-32^ |
| **CMM → Death** | Never | 1 [Reference] | - |  | 1 [Reference] | - |
|  | Former | 1.17 (1.00~1.36) | 0.049 |  | 1.21 (1.06~1.37) | 4.85×10^-3^ |
|  | Current | 1.84 (1.52~2.21) | 1.59×10^-10^ |  | 2.00 (1.72~2.33) | 4.72×10^-19^ |

Note: Never: never smokers, Current: current smokers, FCMD: first cardiometabolic disease, CMM: cardiometabolic multimorbidity.

# Table S7. Estimated AF% and PAF% by sex-specific in ever vs never smokers

| **Association** | **Female and Male** | | |  | **Female** | | |  | **Male** | | |
| --- | --- | --- | --- | --- | --- | --- | --- | --- | --- | --- | --- |
|  | HR (95%CI) | AF% | PAF% |  | HR (95%CI) | AF% | PAF% |  | HR (95%CI) | AF | PAF% |
| Baseline-FCMD | 1.29 (1.29~1.31) | 22.3 | 8.9 |  | 1.31 (1.25~1.37) | 20.9 | 8.5 |  | 1.26 (1.26~1.30) | 23.5 | 9.3 |
| Baseline-T2D | 1.57 (1.57~1.64) | 36.1 | 16.1 |  | 1.56 (1.46~1.67) | 36.0 | 14.5 |  | 1.56 (1.56~1.67) | 35.9 | 18.0 |
| Baseline-CHD | 1.45 (1.45~1.50) | 31.1 | 13.3 |  | 1.57 (1.31~1.88) | 26.4 | 14.7 |  | 1.36 (1.36~1.42) | 36.2 | 12.3 |
| Baseline-Stroke | 1.40 (1.40~1.48) | 28.4 | 11.9 |  | 1.42 (1.29~1.57) | 27.1 | 11.4 |  | 1.37 (1.37~1.49) | 29.8 | 12.6 |
| Baseline-Hypertension | 1.28 (1.28~1.30) | 21.7 | 8.6 |  | 1.29 (1.24~1.34) | 20.8 | 8.0 |  | 1.26 (1.26~1.30) | 22.4 | 9.3 |
| Baseline-Death | 1.51 (1.51~1.57) | 33.9 | 14.9 |  | 1.57 (1.42~1.74) | 31.7 | 14.8 |  | 1.46 (1.46~1.53) | 36.4 | 15.3 |
| FCMD-CMM | 1.75 (1.75~1.82) | 42.8 | 20.3 |  | 1.75 (1.65~1.86) | 42.5 | 18.6 |  | 1.74 (1.74~1.84) | 42.9 | 22.4 |
| T2D-CMM | 1.18 (1.18~1.22) | 15.4 | 5.8 |  | 1.20 (1.13~1.27) | 14.2 | 5.6 |  | 1.17 (1.17~1.22) | 16.4 | 6.1 |
| CHD-CMM | 1.07 (1.07~1.14) | 6.4 | 2.3 |  | 1.09 (0.98~1.20) | 4.8 | 2.5 |  | 1.05 (1.05~1.14) | 7.9 | 1.9 |
| Stroke-CMM | 1.08 (1.08~1.13) | 7.6 | 2.7 |  | 1.07 (1.00~1.15) | 8.2 | 2.2 |  | 1.09 (1.09~1.15) | 6.8 | 3.4 |
| Hypertension-CMM | 1.09 (1.09~1.19) | 8.5 | 3.1 |  | 1.08 (0.96~1.23) | 9.7 | 2.5 |  | 1.11 (1.11~1.24) | 7.8 | 4.0 |
| FCMD-Death | 1.20 (1.20~1.25) | 16.8 | 6.4 |  | 1.22 (1.15~1.31) | 15.5 | 6.3 |  | 1.18 (1.18~1.24) | 18.2 | 6.7 |
| T2D-Death | 1.48 (1.48~1.58) | 32.6 | 14.1 |  | 1.46 (1.32~1.61) | 33.1 | 12.1 |  | 1.49 (1.49~1.63) | 31.3 | 16.2 |
| CHD-Death | 1.48 (1.48~1.68) | 32.2 | 13.9 |  | 1.45 (1.19~1.77) | 33.3 | 12.0 |  | 1.50 (1.50~1.79) | 31.1 | 16.3 |
| Stroke-Death | 1.63 (1.63~1.80) | 38.6 | 17.6 |  | 1.63 (1.40~1.90) | 38.4 | 16.1 |  | 1.62 (1.62~1.84) | 38.8 | 19.6 |
| Hypertension-Death | 1.20 (1.20~1.34) | 16.5 | 6.3 |  | 1.20 (1.02~1.42) | 17.0 | 5.8 |  | 1.20 (1.20~1.41) | 16.9 | 7.4 |
| CMM-Death | 1.49 (1.49~1.59) | 32.8 | 14.2 |  | 1.52 (1.36~1.69) | 30.7 | 13.5 |  | 1.44 (1.44~1.57) | 34.0 | 14.7 |

Note: T2D: type II diabetes, CHD: coronary heart disease, FCMD: first cardiometabolic disease, CMM: cardiometabolic multimorbidity.


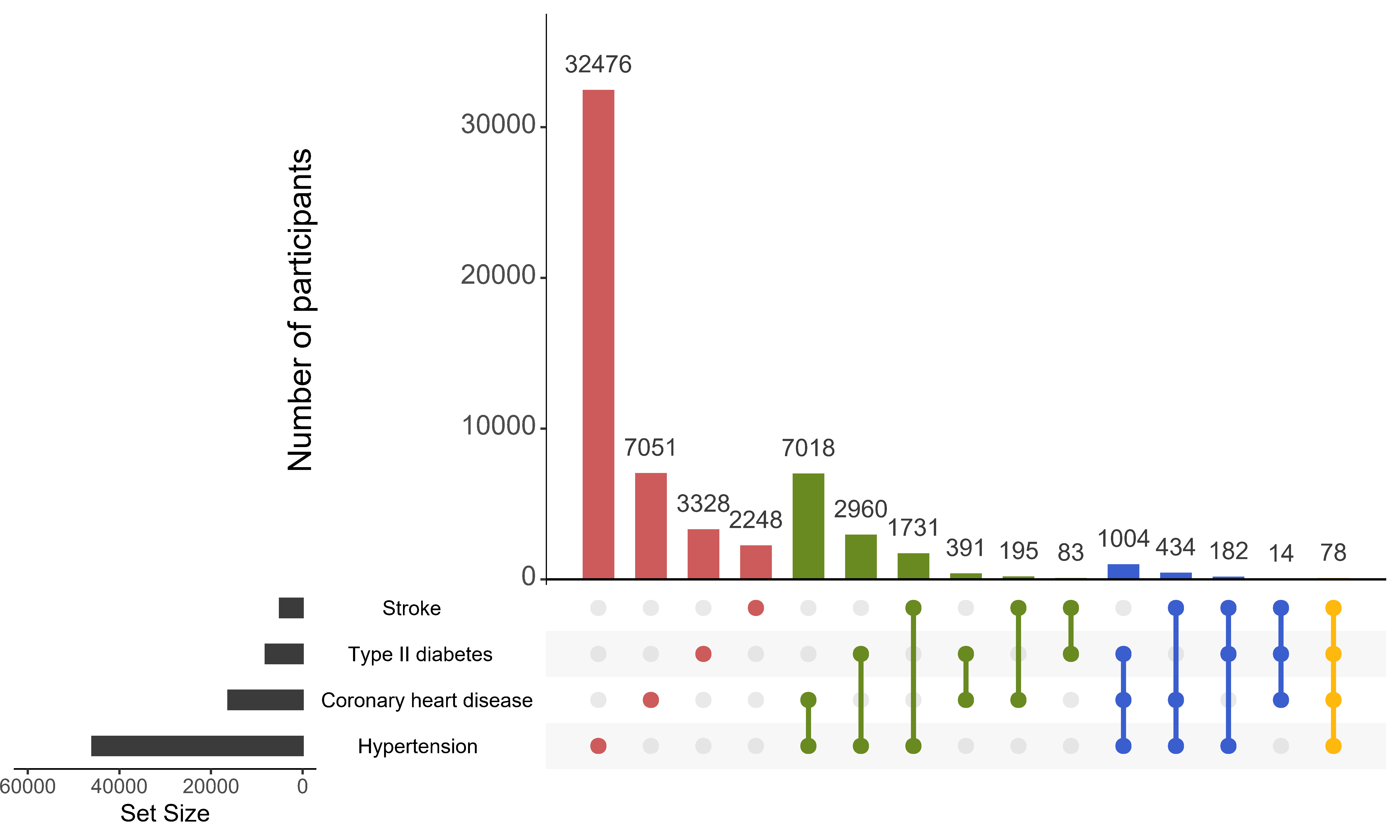


# Figure S1. Upset diagram representing the number of participants for each CMD and different statuses of CMM.



Figure S2. Subgroup analysis of the association of cigarette smoking, smoking cessation with FCMD, CMM, and death stratified by BMI in pattern A and pattern B. Note: FCMD: first cardiometabolic disease; CMM: cardiometabolic multimorbidity.



Figure S3. Subgroup analysis of the association of cigarette smoking, smoking cessation with FCMD, CMM, and death stratified by physical activity in pattern A and pattern B. Note: FCMD: first cardiometabolic disease; CMM: cardiometabolic multimorbidity.



Figure S4. Subgroup analysis of the association of cigarette smoking, smoking cessation with FCMD, CMM, and death stratified by sex in pattern A and pattern B. Note: FCMD: first cardiometabolic disease; CMM: cardiometabolic multimorbidity.





Figure S5. Subgroup analysis of the association of cigarette smoking, smoking cessation with FCMD, CMM, and death stratified by income in pattern A and pattern B. Note: FCMD: first cardiometabolic disease; CMM: cardiometabolic multimorbidity.





Figure S6. Subgroup analysis of the association of cigarette smoking, smoking cessation with FCMD, CMM, and death stratified by drinking status in pattern A and pattern B. Note: FCMD: first cardiometabolic disease; CMM: cardiometabolic multimorbidity.





Figure S7. Subgroup analysis of the association of cigarette smoking, smoking cessation with FCMD, CMM, and death stratified by healthy diet score in pattern A and pattern B. Note: FCMD: first cardiometabolic disease; CMM: cardiometabolic multimorbidity.





Figure S8. Subgroup analysis of the association of cigarette smoking, smoking cessation with FCMD, CMM, and death stratified by TDI median in pattern A and pattern B. Note: FCMD: first cardiometabolic disease; CMM: cardiometabolic multimorbidity.


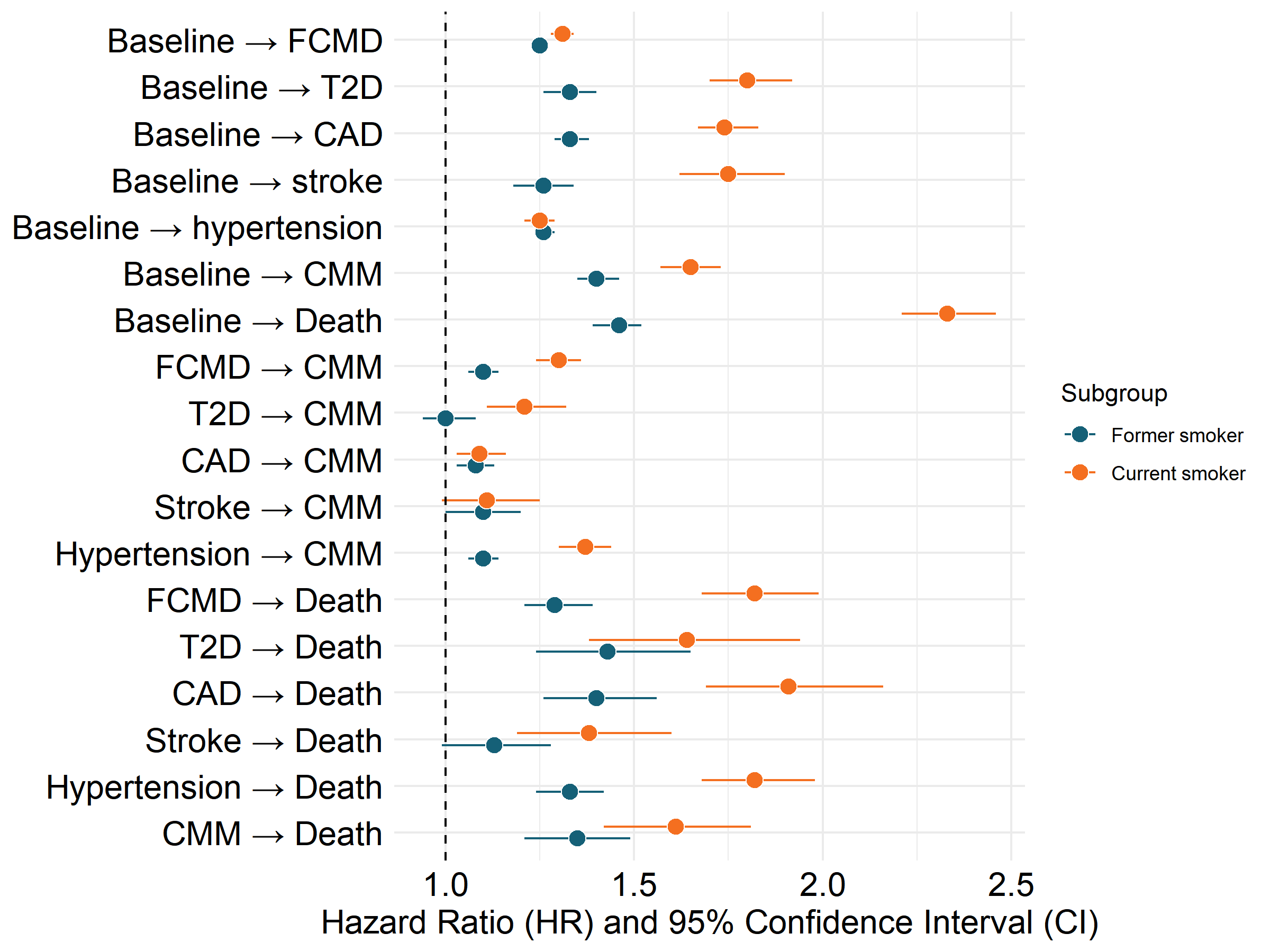


Figure S9. Associations of cigarette smoking, smoking cessation with FCMD, CMM and all-cause mortality in Caucasians with different baseline conditions were analysed after excluding smokers who quit smoking because of illness/disease and smokers who quit on the advice of a physician according to field 6157.


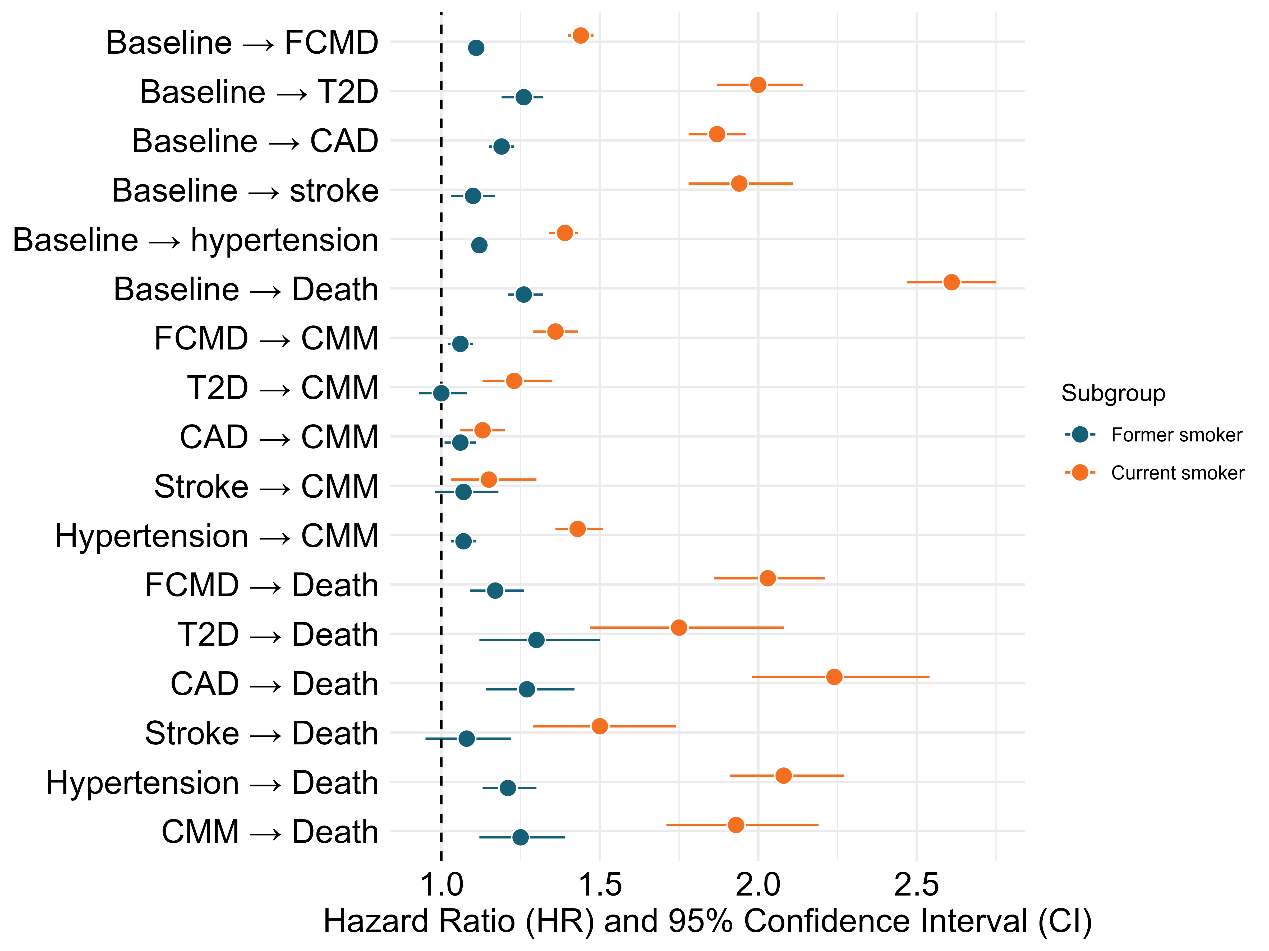


Figure S10. Associations of cigarette smoking, smoking cessation with FCMD, CMM and all-cause mortality in Caucasians with different baseline conditions were analysed after excluding cases/deaths that occurred within 2 years prior to follow-up and participants with duration of smoking cessation less than 1 year.

**References**

1. Office for National Statistics. Median Gross Income, All Households 1977 to Financial Year Ending 2016. 2017. <https://www.ons.gov.uk/peoplepopulationandcommunity/personalandhouseholdfinances/incomeandwealth/adhocs/007767mediangrossincomeallhouseholds1997tofinancialyearending2016>. Accessed Nov 23, 2017.

2. Lee PH, Macfarlane DJ, Lam TH, Stewart SM. Validity of the International Physical Activity Questionnaire Short Form (IPAQ-SF): a systematic review. Int J Behav Nutr Phys Act. 2011, 8:115.

3. Wang M, Zhou T, Song Y, Li X, Ma H, Hu Y, Heianza Y, Qi L. Joint exposure to various ambient air pollutants and incident heart failure: a prospective analysis in UK Biobank. Eur Heart J. 2021, 42(16):1582-1591.

4. Warrington NM, Beaumont RN, Horikoshi M, Day FR, Helgeland Ø, Laurin C, Bacelis J, Peng S, Hao K, Feenstra B *et al*. Maternal and fetal genetic effects on birth weight and their relevance to cardio-metabolic risk factors. Nat Genet. 2019, 51(5):804-814.

5. Jin S, Wang T, Wenying C, Wu Y, Huang S, Zeng P. Maternal and fetal origins of offspring blood pressure: statistical analysis using genetic correlation and genetic risk score-based Mendelian randomization. Int J Epidemiol. 2023:dyad034.
